# Adverse Childhood Experiences Promote Increased and Selective Caregiving in Adulthood

**DOI:** 10.3390/ijerph23020213

**Published:** 2026-02-09

**Authors:** Ray M. Merrill

**Affiliations:** Department of Public Health, College of Life Sciences, Brigham Young University, Provo, UT 84602, USA; ray_merrill@byu.edu; Tel.: +1-(801)-422-9788

**Keywords:** adverse childhood experiences, BRFSS, caregiver, emotional parentification, instrumental parentification

## Abstract

**Highlights:**

**Public health relevance—How does this work relate to a public health issue?**

- Adverse Childhood Experiences (ACEs) can prompt parentification, which often extends to a propensity toward providing caregiving as adults.
- All types of ACEs can significantly increase providing caregiving as adults.

**Public health significance—Why is this work of significance to public health?**

- While ACE-induced parentification may foster some positive characteristics, like empathy, responsibility, life skills, coping skills, and autonomy, it may result in adults becoming compulsive caretakers in relationships, putting others’ needs first, and feeling their self-worth depends on “being needed.”
- Adult caregivers are more likely to provide regular care for a spouse, child, sibling, friend or non-relative versus a parent as ACEs increase.

**Public health implications—What are the key implications or messages for practitioners, policy makers and/or researchers in public health?**

- There are both positive and negative aspects of ACE-induced caregiving, which should be recognized and balanced.
- ACE-driven caregivers should receive counseling to help them better understand motives and ways they can protect their own health and wellbeing, and that of others.

**Abstract:**

Adverse childhood experiences (ACEs) prompt parentification, which is related to providing regular caregiving as adults. This study examines the association between the number and types of ACEs and caregiving as adults, and to whom caregiving is extended. Analyses were based on 90,666 adults from 13 states in the U.S. in 2020–2024 and involved binomial and multinomial logistic regression, adjusted for selected covariates. Approximately 21% of participants provided regular care and 66% had ≥1 ACEs. Each of 11 ACEs considered was positively associated with providing regular care. As the number of types of ACEs increased, the odds of providing regular care increased. The strongest ACE predictor of providing regular care was living with a parent or adult who was depressed, mentally ill, or suicidal, and the weakest was when the parents were divorced, separated, or an unmarried couple. Among those receiving regular care, if the caregiver had more ACEs versus less, they were significantly less likely to care for a parent or grandparent but more likely to care for a spouse, child, sibling, or friend or non-relative.

## 1. Introduction

Parentification involves role reversal in which a child assumes developmentally inappropriate parent or adult roles and responsibilities for their age [1,2]. Classifications of parentification are emotional or instrumental, where the child assumes adult roles that can include confidant, counselor, emotional supporter, household earner, self-carer, caregiver, family-navigator, and language and cultural broker [1,3,4,5]. Dariotis et al. conducted a systematic review and meta-analysis to identify 95 studies from six continents on parentification, which showed that parentified youth may experience positive outcomes, negative outcomes, and to mitigate further trauma, employ protective strategies [1]. Positive outcomes include enhanced empathy, responsibility, life skills, coping skills, giving, and autonomy, negative outcomes include internalizing problems (e.g., anxiety, depression), externalizing problems (e.g., sexual risk taking, substance use), and compromised physical health (e.g., poor nutrition, physical abuse), and protective strategies may involve seeking social support, disclosing worries, and attending religious services [1,6].

Parentification tends to result from the primary caregiver failing to fulfill their responsibilities, resulting in neglect or maltreatment. Some common sources of youth role reversal and role overload include parental loss (e.g., divorce, incarceration, or death), parental mental or physical illness, disability, or crisis, dysfunctional family dynamics (e.g., domestic partner violence), and parents who were parentified themselves expecting the same of their child [1,7]. The strong association between parentification and caregiving stems from a child taking on the role of caregiver, which is generally reserved for adults. Several studies show that parentification significantly increases caretaking behavior, sometimes resulting in adults becoming compulsive caretakers in relationships, putting other people’s needs first, and feeling their self-worth corresponds with “being needed,” even when the care they provide is unnecessary [8,9,10]. Adverse childhood experiences (ACEs) in general have been associated with increased the risk of parentification [11,12,13,14,15,16,17,18,19,20,21].

The extent that specific ACEs are associated with caregiving as adults is not well understood, although caregiving behavior associated with parentification often extends into adulthood; furthermore, it is unclear whether ACEs result in selective caregiving. The purpose of the current study is to examine the association between the number and type of ACEs and providing regular care as adults, and to whom they provide that care. We hypothesize a positive association between the number of types of ACEs and caregiving as adults, that all ACEs positively associate with providing regular care among adults, and that, among those who provide regular care, providing it will be similar among spouses, children, siblings, friends or non-relatives versus parents as the number of types of ACEs increases.

## 2. Materials and Methods

### 2.1. Data

Data was obtained from the 2020–2024 Behavior Risk Factor Surveillance System (BRFSS). The BRFSS is a national system of health-related telephone surveys, which collects data from residents in all 50 states, the District of Columbia, and 3 U.S. territories about health behaviors, chronic health conditions, and preventive service utilization. BRFSS surveys employ a cross-sectional design with standardized questionnaires. Annually, over 400,000 adult surveys (ages 18 and older) are completed, using random probability sampling on landlines and cell phones. BRFSS surveys consist of 3 components: (1) standard core questions, (2) optional module questions on specific topics (e.g., cannabis use), and (3) area-added questions [22]. Median response rates for all participating states and territories were 45% (2020), 44% (2021), 45% (2022), 47% (2023), and 46% (2024) [23,24,25,26,27].

Not all BRFSS areas included questions about both ACEs and provision of care. For those areas that did, these questions were not consistently asked across years. Data analysis involved those 13 BRFSS areas in the U.S. that included questions about both ACEs and provision of care or assistance for a friend or family member who had a health problem or disability in one or more years during 2020–2024 (Table 1). The total number of participants was 91,193. Although BRFSS does not require informed consent for participation, and individuals are informed that they do not need to participate and can answer any questions, verbal consent is obtained during initial contact. A description of the BRFSS survey design, questionnaires, and data collection methods is available elsewhere [28].

### 2.2. Measures

Providing regular care for a friend or family member was based on the question: “During the past 30 days, did you provide regular care or assistance to a friend or family member who has a health problem or disability?” A small number (n = 527, 0.58%) did not know or refused to indicate whether they provided care or assistance for someone. A sensitivity analysis showed that deleting the missing values versus combining them with those who provided regular care or combining them with those who did not provide regular care only affected the results to the nearest tenth place after the decimal in the tables. Hence, we deleted the missing values for the provision of care variable, leaving 90,666 for analysis. Those who responded “Yes” to the question about providing regular care were further asked: “What is his or her relation to you?” Possible responses are parent, stepparent, or parent-in-law; grandparent, step grandparent, or grandparent-in-law; spouse or partner; child or stepchild, grandchild or step grandchild; sibling, stepsibling, sibling-in-law, or other relative; and friend or non-relative. Of the original sample, 212 (0.23%) were not asked this question. 

Demographic variables appear in Table 2; selected mental and physical health conditions, obesity, and health-risk behaviors appear in Table 3; and 11 ACE items appear in Table 4.

### 2.3. Statistical Analysis

Descriptive statistics included frequencies and percentages (standard errors). Estimates were determined by taking the survey stratum, primary sampling units, and sampling weights into consideration. We used binomial logistic regression to estimate the odds of providing regular care according to demographics, mental and physical health conditions, difficulty making decisions, depression, obesity, and health-risk behaviors. It also estimated the odds of providing regular care separately for each ACE. Multinomial logistic regression estimated the odds of ≥5, 3–4, 1–2, or 0 ACEs according to demographics and mental and physical health conditions, difficulty making decisions, depression, obesity, and health-risk behaviors. We adjusted the odds ratios for selected covariates. Type 3 analysis of effects in logistic regression evaluated the significance of each ACE in the presence of all other covariates in predicting proving regular care. We presented confidence intervals associated with the odds ratios, indicating statistical significance when they did not overlap 1. All statistical tests were two-sided, with a significance level of 0.05. Analyses were conducted using Statistical Analysis System (SAS) software, version 9.4 (SAS Institute Inc., Cary, NC, USA, 2016).

## 3. Results

Demographic characteristics of the participants are presented in Table 2. Most participants were 45 years or older (58.12%), women (52.05%), non-Hispanic (NH) Whites (64.25%), married/cohabitating (55.56%), had some college or a college degree (60.38%), and had an annual household income <$100,000 (65.19%). The highest number of U.S. areas asking questions about both providing regular care or assistance and ACEs was in 2021.

**Table 2:** Odds of providing regular care and of ACEs by demographics.

			Regular Care or Assistance		ACEs	
	No.	% (SE) *	% (SE) *	Odds Ratio (95% CI) *^†^	Mean Number (SE) *	Odds Ratio (95% CI) *^†^
Age						
18–24	4779	10.89 (0.23)	15.65 (0.98)	1.00	2.64 (0.07)	1.00
25–34	8348	15.14 (0.23)	16.48 (0.62)	1.01 (0.85–1.20)	2.76 (0.04)	0.78 (0.59–1.04)
35–44	11,006	15.90 (0.24)	20.04 (0.64)	1.33 (1.11–1.58)	2.39 (0.04)	0.58 (0.44–0.77)
45–54	12,863	15.74 (0.23)	23.76 (0.68)	1.68 (1.41–2.01)	2.19 (0.04)	0.43 (0.33–0.57)
55–64	16,651	16.98 (0.23)	27.26 (0.72)	1.96 (1.63–2.35)	1.86 (0.03)	0.26 (0.19–0.35)
≥65	37,019	25.35 (0.22)	21.61 (0.39)	1.39 (1.18–1.65)	1.24 (0.02)	0.16 (0.12–0.20)
Sex						
Men	41,006	47.92 (0.31)	18.02 (0.36)	1.00	1.86 (0.02)	1.00
Women	49,660	52.08 (0.31)	24.19 (0.37)	1.44 (1.35–1.53)	2.24 (0.02)	1.27 (1.14–1.41)
Race/ethnicity						
NH White	68,648	64.31 (0.30)	22.26 (0.27)	1.00	2.05 (0.02)	1.00
NH Black	10,123	15.17 (0.25)	22.50 (0.76)	1.03 (0.94–1.13)	2.01 (0.04)	0.82 (0.71–0.95)
NH Other	4866	7.04 (0.16)	19.18 (0.90)	0.87 (0.77–0.98)	2.29 (0.06)	1.35 (1.12–1.64)
Hispanic	5227	11.73 (0.25)	15.62 (1.25)	0.70 (0.58–0.85)	2.05 (0.07)	0.89 (0.66–1.18)
Unknown	1802	1.76 (0.07)	18.15 (1.31)	0.83 (0.69–1.00)	1.95 (0.09)	1.09 (0.81–1.46)
Marital Status						
Mar/Cohab	49,849	55.57 (0.32)	22.63 (0.33)	1.00	1.90 (0.02)	1.00
Previously M	25,877	21.46 (0.24)	20.88 (0.53)	0.74 (0.69–0.81)	2.07 (0.03)	1.22 (1.08–1.37)
Never M	14,272	22.25 (0.29)	18.24 (0.64)	0.90 (0.80–1.01)	2.44 (0.04)	0.98 (0.81–1.19)
Unknown	668	0.72 (0.05)	16.52 (2.07)	0.75 (0.56–1.02)	1.72 (0.17)	0.83 (0.51–1.34)
Education						
<HS	6032	11.04 (0.24)	18.32 (0.86)	1.00	2.29 (0.06)	1.00
HS	23,343	28.20 (0.30)	20.81 (0.52)	1.15 (1.01–1.32)	2.16 (0.03)	0.74 (0.60–0.91)
Some college	25,998	32.09 (0.29)	23.54 (0.50)	1.32 (1.15–1.51)	2.21 (0.03)	0.77 (0.62–0.95)
College	34,995	28.34 (0.25)	20.26 (0.36)	1.13 (0.99–1.29)	1.70 (0.02)	0.60 (0.49–0.74)
Unknown	298	0.32 (0.03	13.71 (2.60)	0.85 (0.54–1.34)	1.07 (0.14)	0.29 (0.15–0.57)
Income						
<50 K	33,899	37.09 (0.31)	23.85 (0.45)	1.00	2.33 (0.03)	1.00
50 K to <100 K	24,375	28.27 (0.29)	21.21 (0.54)	0.78 (0.71–0.85)	2.03 (0.03)	0.76 (0.67–0.87)
100 K to <200 K	12,123	12.15 (0.15)	19.87 (0.53)	0.65 (0.59–0.71)	2.01 (0.03)	0.80 (0.69–0.93)
≥200 K	3382	3.95 (0.10)	17.27 (0.95)	0.53 (0.46–0.62)	1.75 (0.05)	0.52 (0.41–0.66)
Unknown	16,887	18.55 (0.25)	17.76 (0.56)	0.66 (0.60–0.73)	1.66 (0.03)	0.61 (0.53–0.70)
Year						
2020	15,666	27.57 (0.21)	19.51 (0.72)	1.00	1.87 (0.04)	1.00
2021	44,769	35.27 (0.17)	21.44 (0.30)	1.15 (1.05–1.26)	2.02 (0.02)	0.92 (0.80–1.07)
2022	13,013	13.41 (0.14)	20.32 (0.49)	1.14 (1.03–1.27)	2.15 (0.03)	1.23 (1.03–1.46)
2023	9692	12.06 (0.11)	24.77 (0.65)	1.43 (1.28–1.59)	2.35 (0.04)	1.24 (1.04–1.47)
2024	7526	11.68 (0.17)	22.06 (0.73)	1.29 (1.14–1.46)	2.21 (0.05)	1.41 (1.16–1.71)

Data source: BRFSS, 2020–2024. SE: Standard Error; CI: Confidence Interval; NH: Non-Hispanic. * Weighted estimates, based on the complex sampling design. ^†^ Binomial logistic regression adjusted for age, sex, race/ethnicity, marital status, education, annual household income, and year. ^‡^ Multinomial logistic regression adjusted for age, sex, race/ethnicity, marital status, education, annual household income, and year. The outcome in this model is ≥5 vs. 3–4 vs. 1–2 vs. 0 ACEs.

A total of 19,691 participants (21% weighted) reported providing regular care. Providing regular care was significantly greater in older age groups, women, NH White participants and NH Black participants, married/cohabitating, those with some college and lower income. ACEs were more common in younger age groups, women, NH White participants, previously married, and in those with lower education and income.

As shown in Table 3, health and behavior variables were generally significantly associated with both providing regular care and having ACEs. Specifically, providing regular care was significantly greater among those reporting a greater number of poor mental health days, poor physical health days, difficulty making decisions, chronic depression, obesity, and being a current or former smoker. The provision of regular care was not significantly associated with heavy drinking. Having ACEs was significantly more common in all the health and behavior variables, including heavy drinking.

**Table 3:** Odds of providing regular care or assistance and of ACEs by selected mental and physical health conditions, obesity, and health-risk behaviors.

			Regular Care or Assistance		ACEs	
	No.	% (SE) *	% (SE) *	Odds Ratio (95% CI) *^†^	Mean Number (SE) *	Odds Ratio (95% CI) *^‡^
Poor Mental Health Days						
0 days not Gd	55,513	57.49 (0.31)	18.13 (0.33)	1.00	1.47 (0.02)	1.00
1–13 days not Gd	21,179	25.23 (0.27)	23.85 (0.55)	1.36 (1.26–1.47)	2.52 (0.03)	1.62 (1.53–1.72)
14–30 days not Gd	12,279	15.35 (0.22)	28.16 (0.66)	1.50 (1.36–1.66)	3.52 (0.05)	2.00 (1.84–2.17)
Unknown	1695	1.93 (0.09)	18.20 (1.78)	1.07 (0.84–1.36)	1.87 (0.10)	1.02 (0.86–1.21)
Poor Physical Health Days						
0 days not Gd	56,351	63.07 (0.30)	19.13 (0.33)	1.00	1.78 (0.02)	1.00
1–13 days not Gd	20,126	22.68 (0.26)	24.88 (0.57)	1.17 (1.09–1.26)	2.48 (0.04)	1.24 (1.17–1.31)
14–30 days not Gd	12,178	12.17 (0.19)	24.69 (0.65)	0.92 (0.84–1.00)	2.71 (0.04)	1.19 (1.11–1.29)
Unknown	2011	2.09 (0.09)	19.11 (1.62)	0.95 (0.77–1.17)	1.97 (0.10)	1.07 (0.91–1.26)
Difficulty Making Decisions						
Yes	10,838	13.36 (0.22)	28.65 (0.83)	1.31 (1.18–1.44)	3.60 (0.06)	1.63 (1.51–1.77)
No	79,203	85.98 (0.22)	20.05 (0.27)	1.00	1.81 (0.01)	1.00
Unknown	625	0.66 (0.04)	24.68 (2.73)	1.28 (0.95–1.73)	2.50 (0.16)	1.34 (1.08–1.67)
Depression						
Yes	18,486	20.76 (0.24)	28.12 (0.63)	1.15 (1.06–1.25)	3.46 (0.04)	1.85 (1.74–1.97)
No	71,703	78.65 (0.25)	19.40 (0.28)	1.00	1.68 (0.01)	1.00
Unknown	477	0.59 (0.06)	23.37 (6.02)	1.17 (0.62–2.21)	2.62 (0.20)	1.60 (1.20–2.12)
Obesity						
Yes	29,752	32.26 (0.29)	23.92 (0.44)	1.12 (1.05–1.19)	2.29 (0.02)	1.15 (1.10–1.21)
No	55,289	61.12 (0.31)	20.43 (0.34)	1.00	1.97 (0.02)	1.00
Unknown	5625	6.62 (0.18)	15.55 (0.85)	0.74 (0.64–0.85)	1.67 (0.06)	0.78 (0.69–0.87)
Smoking						
Current	12,013	14.61 (0.24)	28.24 (0.84)	1.51 (1.37–1.68)	2.94 (0.05)	1.85 (1.72–1.99)
Past	25,755	25.12 (0.25)	21.58 (0.49)	1.05 (0.98–1.13)	2.24 (0.03)	1.63 (1.54–1.72)
Never	52,278	59.61 (0.31)	19.38 (0.32)	1.00	1.77 (0.02)	1.00
Unknown	620	0.65 (0.05)	19.49 (2.68)	1.05 (0.74–1.47)	1.69 (0.17)	1.09 (0.83–1.44)
Heavy Drinker						
Yes	5465	6.23 (0.15)	21.63 (0.94)	0.92 (0.81–1.03)	2.68 (0.06)	1.30 (1.19–1.42)
No	83,506	91.75 (0.18)	21.31 (0.27)	1.00	2.02 (0.02)	1.00
Unknown	1695	2.02 (0.09)	16.29 (1.55)	0.82 (0.65–1.03)	2.00 (0.10)	1.04 (0.88–1.22)

Data source: BRFSS, 2020–2024. SE: Standard Error; CI: Confidence Interval. * Weighted estimates, based on the complex sampling design. ^†^ Binary logistic regression estimated odds rated were adjusted for age, sex, race/ethnicity, marital status, education, annual household income, year, and the variables in the table. ^‡^ Multinomial logistic regression estimated odds ratios were adjusted for age, sex, race/ethnicity, marital status, education, annual household income, year, and the variables in the table. The outcome measure in this model is ≥5 vs. 3–4 vs. 1–2 vs. 0 ACEs. Heavy drinker is defined as an adult man having more than 14 drinks per week and an adult women having more than 7 drinks per week.

Distributions of specific ACEs appear in Table 4. The most reported type of ACEs was having parents who separated or divorced (31% weighted) and having a parent or adult in the home swear at you, insult you, or put you down more than once (30% weighted). The least common ACE involved anyone at least 5 years older than you or an adult forcing you to have sex (2% for “once” and 4% for “more than once” weighted).

The odds of providing regular care were estimated for each ACE item (Table 4), adjusting for the demographic, health, and behavior variables. A significant positive association occurred in each model. Living with anyone who was depressed, mentally ill, or suicidal had the largest effect and having parents who were divorced had the smallest effect. the odds of providing regular care were also estimated simultaneously for all the ACE items included in the model, along with the demographic, health, and behavior variables. A significant direct positive association remained between ACE items and providing regular care for those items involving living with anyone who was depressed, mentally ill, or suicidal; living with anyone who was a problem drinker or alcoholic; living with anyone who served time or was sentenced to serve time in prison, jail, or other correctional facility; having parents or adults in their home who physically hurt each other; and having anyone, at least 5 years older than you or an adult, ever touch you sexually or force you to have sex. Other ACE items may have become statistically insignificant because their effect on providing regular care was mediated by another ACE item included in the model.

**Table 4:** Odds of providing regular care or assistance by specific ACE items.

				Regular Care or Assistance			
	No.	% (SE) *	% (SE) *	Odds Ratio (95% CI) *^†^	Type 3 F, (Pr > F)	Odds Ratio (95% CI) *^‡^	Type 3 F, (Pr > F)
Did you live with anyone who was depressed, mentally ill, or suicidal?							
Yes	16,543	19.83 (0.23)	27.68 (0.57)	1.39 (1.29–1.50)	34.97	1.18 (1.08–1.30)	7.68
No	72,100	77.85 (0.24)	19.55 (0.30)	1.00	<0.0001	1.00	0.0005
Unknown	2023	2.31 (0.08)	22.61 (1.47)	1.22 (1.02–1.45)		1.28 (1.03–1.58)	
Did you live with anyone who was a problem drinker or alcoholic?							
Yes	21,728	24.67 (0.27)	26.73 (0.57)	1.33 (1.24–1.42)	30.12	1.11 (1.02–1.20)	3.45
No	67,322	73.45 (0.27)	19.41 (0.29)	1.00	<0.0001	1.00	0.0318
Unknown	1616	1.88 (0.08)	20.33 (1.61)	1.06 (0.86–1.30)		0.92 (0.65–1.32)	
Did you live with anyone who used illegal street drugs or who abused prescription medications?							
Yes	9265	12.71 (0.21)	27.81 (0.87)	1.41 (1.28–1.56)	24.55	1.09 (0.98–1.22)	2.09
No	79,637	85.20 (0.22)	20.23 (0.27)	1.00	<0.0001	1.00	0.1239
Unknown	1764	2.09 (0.08)	22.15 (1.62)	1.17 (0.97–1.42)		1.28 (0.90–1.80)	
Did you live with anyone who served time or was sentenced to serve time in a prison, jail, or other correctional facility?							
Yes	6794	10.17 (0.21)	27.49 (1.06)	1.46 (1.30–1.64)	3.79	1.18 (1.04–1.34)	3.14
No	82,314	87.89 (0.22)	20.54 (0.26)	1.00	0.0226	1.00	0.0433
Unknown	1558	1.94 (0.08)	19.56 (1.68)	1.02 (0.83–1.28)		0.93 (0.59–1.46)	
Were your parents separated or divorced?							
Yes	23,491	31.36 (0.30)	22.69 (0.51)	1.11 (1.03–1.19)	3.63	0.95 (0.88–1.02)	2.09
No	63,800	63.89 (0.31)	20.53 (0.30)	1.00	0.0123	1.00	0.1239
Couple not M	1390	2.19 (0.10)	24.34 (2.19)	1.21 (0.94–1.55)		1.03 (0.80–1.33)	
Unknown	1985	2.56 (0.11)	18.12 (1.47)	0.90 (0.73–1.11)		0.70 (0.50–0.97)	
How often did your parents or adults in your home ever slap, hit, kick, punch or beat each other up?							
0	73,101	78.63 (0.25)	19.75 (0.28)	1.00	24.61	1.00	4.49
1	3474	4.31 (0.12)	24.25 (1.20)	1.27 (1.11–1.45)	<0.0001	1.16 (1.01–1.33)	0.0037
≥2	10,880	13.09 (0.21)	29.16 (0.83)	1.46 (1.33–1.59)		1.19 (1.07–1.32)	
Unknown	3211	3.96 (0.12)	21.22 (1.23)	1.12 (0.96–1.31)		1.10 (0.88–1.36)	
Not including spanking (before age 18), how often did a parent or adult in your home ever hit, beat, kick, or physically hurt you in any way? Was it—							
0	67,371	72.32 (0.28)	19.95 (0.30)	1.00	9.02	1.00	0.52
1	5332	5.96 (0.14)	21.96 (0.93)	1.05 (0.94–1.18)	<0.0001	0.95 (0.84–1.07)	0.6677
≥2	15,239	18.37 (0.24)	26.22 (0.67)	1.25 (1.15–1.35)		0.96 (0.87–1.06)	
Unknown	2724	3.35 (0.11)	20.10 (1.31)	1.08 (0.91–1.28)		1.07 (0.83–1.39)	
How often did a parent or adult in your home ever swear at you, insult you, or put you down?							
0	57,897	60.75 (0.30)	19.19 (0.32)	1.00	18.65	1.00	1.45
1	4465	5.15 (0.14)	19.60 (1.01)	1.02 (0.89–1.16)	<0.0001	0.97 (0.85–1.11)	0.2256
≥2	25,107	30.32 (0.29)	25.73 (0.52)	1.30 (1.22–1.40)		1.09 (0.99–1.20)	
Unknown	3197	3.79 (0.12)	20.23 (1.20)	1.08 (0.92–1.25)		1.02 (0.81–1.28)	
How often did anyone, at least 5 years older than you or an adult, ever touch you sexually?							
0	77,090	83.93 (0.23)	19.77 (0.27)	1.00	19.31	1.00	2.72
1	3669	4.23 (0.14)	28.95 (1.76)	1.38 (1.15–1.64)	<0.0001	1.24 (1.02–1.52)	0.0426
≥2	6865	8.16 (0.16)	32.18 (0.99)	1.44 (1.30–1.60)		1.20 (1.03–1.40)	
Unknown	3042	3.69 (0.12)	20.23 (1.20)	1.06 (0.91–1.23)		1.06 (0.71–1.58)	
How often did anyone, at least 5 years older or an adult, try to make you touch them sexually?							
0	79,827	86.73 (0.21)	20.16 (0.28)	1.00	7.59	1.00	0.12
1	2772	3.38 (0.12)	27.65 (1.49)	1.26 (1.08–1.47)	<0.0001	1.00 (0.82–1.21)	0.9465
≥2	5009	6.16 (0.14)	32.37 (1.07)	1.43 (1.28–1.60)		1.05 (0.88–1.24)	
Unknown	3058	3.73 (0.13)	21.90 (1.49)	1.05 (0.89–1.25)		0.95 (0.61–1.48)	
How often did anyone at least 5 years older than you or an adult, force you to have sex?							
0	83,002	90.45 (0.19)	20.37 (0.26)	1.00	14.97	1.00	2.63
1	1531	2.03 (0.11)	32.73 (3.00)	1.55 (1.16–2.06)	<0.0001	1.32 (0.97–1.79)	0.0485
≥2	3054	3.70 (0.11)	35.74 (1.61)	1.59 (1.37–1.85)		1.30 (1.06–1.60)	
Unknown	3079	3.81 (0.12)	21.52 (1.29)	1.04 (0.89–1.22)		1.01 (0.68–1.49)	

Data source: BRFSS, 2020–2024. SE: Standard Error; CI: Confidence Interval. Type 3 analysis of effects was used to indicate the statistical significance of each variable in the presence of all other variables in the model. * Weighted estimates, based on the complex sampling design. ^†^ A separate model was run for each ACE variable, estimated using logistic regression, adjusting for age, sex, race/ethnicity, marital status, education, annual household income, year, poor mental health days, poor physical health days, difficulty making decisions, depression, obesity, smoking status, heavy drinking status. ^‡^ A single model was estimated for all the ACE items simultaneously using logistic regression, adjusting for age, sex, race/ethnicity, marital status, education, annual household income, year, poor mental health days, poor physical health days, difficulty making decisions, depression, obesity, smoking status, and heavy drinking status.

**Figure 1:**
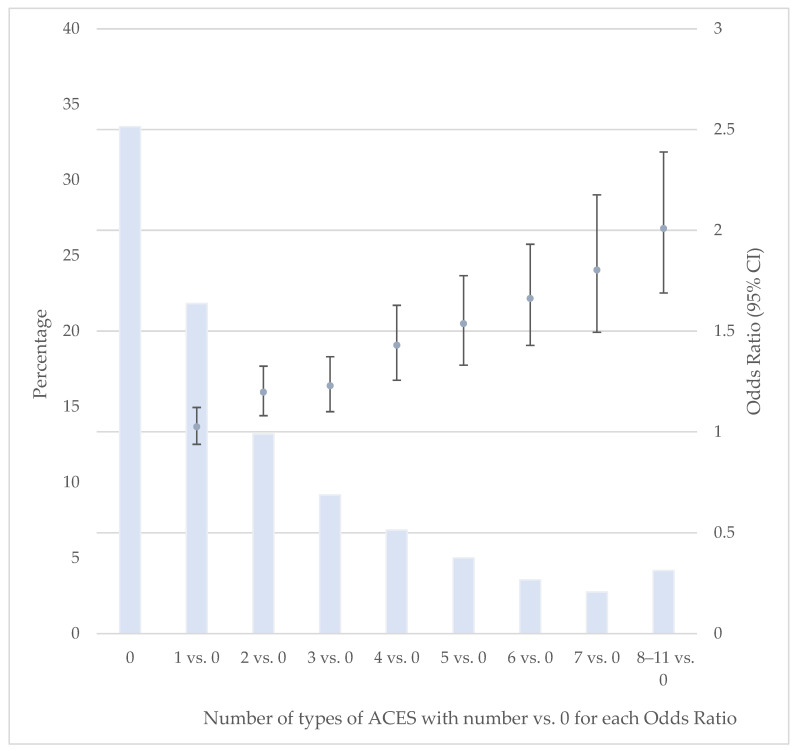
Odds of Providing Regular Care or Assistance by Number of ACEs. Data source: BRFSS, 2020–2024. CI: Confidence Interval. Odds ratios were weighted, based on the complex sampling design, and adjusted for age, sex, race/ethnicity, marital status, education, annual household income, year, poor mental health days, poor physical health days, difficulty making decisions, depression, obesity, smoking status, and heavy drinking status.

Approximately 34% of participants reported no ACEs, 22% reported 1 ACE, and 44% reported 2 or more ACEs. The odds of providing regular care consistently increased with more ACEs (vs. 0), after adjusting for selected covariates (Figure 1). Statistical significance occurred with ≥2 ACEs.

**Figure 2:**
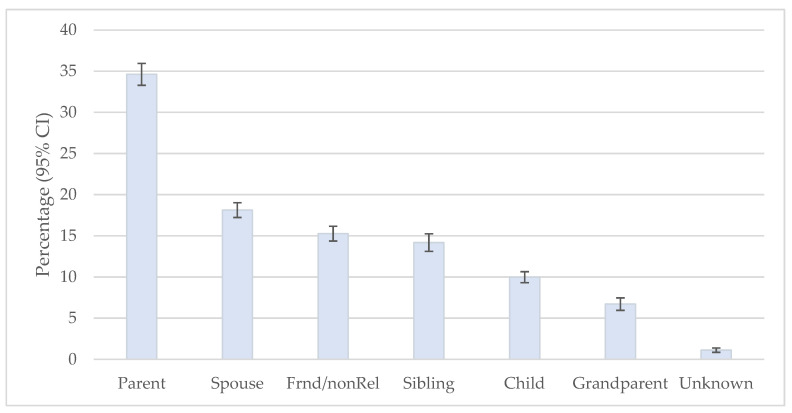
Distribution of Providing Regular Care for Specific Groups. Data source: BRFSS, 2020–2024. CI: Confidence Interval. Weighted estimates, based on the complex sampling design.

Among those participants providing regular care, the percentage doing so for specific groups is presented in Figure 2. Providing regular care for a parent, stepparent, or parent-in-law occurred most often. Providing regular care for any of the other groups occurred about half or less often. The odds of providing regular care for group X versus a parent, stepparent, or parent-in-law by number of ACEs appear in Table 5. The odds of providing regular care for each of the groups tend to be more common than for a parent, stepparent, or parent-in-law as the number of ACEs increases.

**Figure 3:**
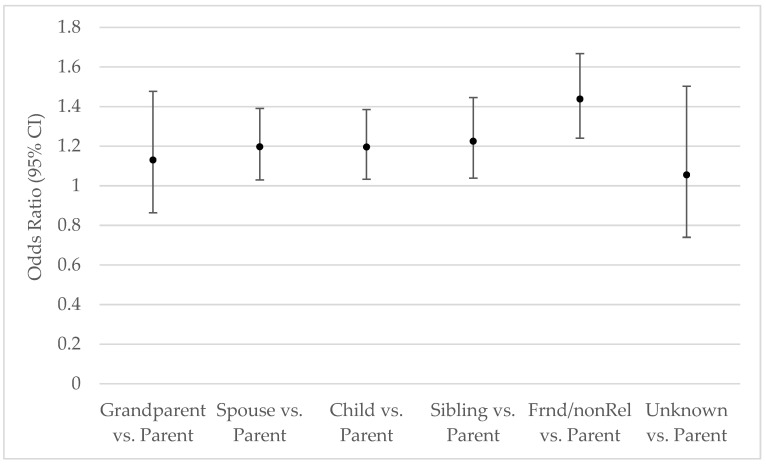
Odds of More ACEs vs. Less for Each Group Compared with the Parent Group. Data source: BRFSS, 2020–2024. CI: Confidence Interval. Multinomial logistic regression estimated odds ratios were weighted, based on the complex sampling design, and adjusted for age, sex, race/ethnicity, marital status, education, income, year, poor mental health days, poor physical health days, difficulty making decisions, depression, obesity, smoking status, and heavy drinking status.

Among those participants providing regular care, Figure 3 shows that those with a higher number of types of ACEs were significantly more likely to care for a spouse, child, sibling, other relative, friend, or non-relative compared with a parent, stepparent, or parent-in-law.

**Figure 4:**
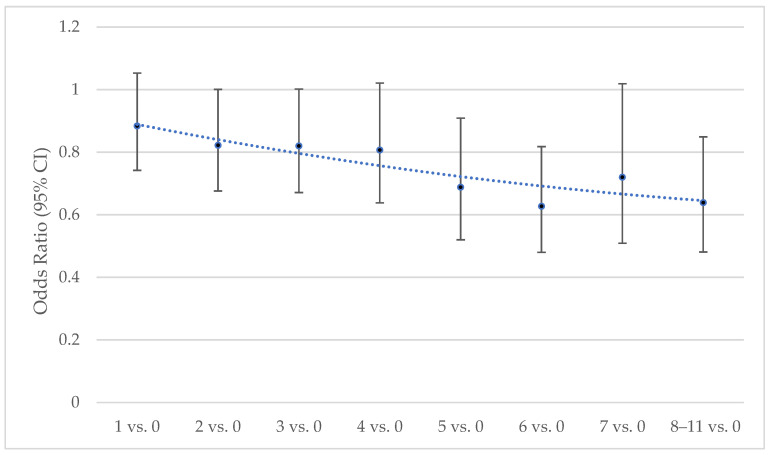
Odds of Providing Regular care for a Parent, Stepparent, or Parent-in-Law versus Care for Another Group by Number of Types of ACEs. Data source: BRFSS, 2020–2024. CI: Confidence Intervale. The trendline was estimated from a second order polynomial regression model. Binary logistic regression estimated odds ratios were weighted, based on the complex sampling design, and adjusted for age, sex, race/ethnicity, marital status, education, income, year, poor mental health days, poor physical health days, difficulty making decisions, depression, obesity, smoking status, and heavy drinking status.

The lower level of providing regular care for a parent, stepparent, or parent-in-law compared with the other groups (not including unknown) is presented by the number of types of ACEs in Figure 4. A significant negative dose–response relationship appears.

## 4. Discussion

This study assessed three hypotheses. First, we hypothesized that a positive association exists between the number of types of ACEs and providing regular care as adults. The results support this hypothesis. After adjustment for covariates, there remained a significant positive dose–response relationship between number of types of ACEs and providing regular care. This is consistent with parentification due to ACEs having a persistent effect on caretaking behavior into adulthood. The literature maintains the idea that ACEs increase the probability of parentification [11,12,13,14,15,16,17,18,19,20,21], with children experiencing parentification often carrying these patterns into adulthood [7,8,9]. For example, parentified adults may become caregiving partners themselves, seeking out needy or emotionally unavailable partners because of conditioning to prioritize other peoples’ needs above their own [1,2,3,4,5]. Thompson et al. found that they may struggle to set boundaries, in fear of rejection or abandonment [11]. Castro et al. referred to the “impostor phenomenon” in which adults who experienced parentification as children had underlying feelings of shame, unworthiness, or fraudulence despite experienced successes, thereby causing them to compensate for perceived deficiencies by working unreasonably hard to satisfy others [29]; yet, some research has shown that parentification in some circumstances and degrees may be positive, promoting the development of empathy, responsibility, life skills, coping skills, giving, and autonomy [1,6]. In a study of Black college students Gilford found an association between parentification and resilience, which together predicted a higher degree of academic motivation [30].

Second, we hypothesized that each ACE item would positively associate with providing regular care among adults. Indeed, each of the ACE items was significantly positively associated with providing care after adjusting for the demographic, health, and behavior variables, as consistent with previous research [11,12,13,14,15,16,17,18,19,20,21]. Running a single model with all the ACE, demographic, health, and behavior variables estimated simultaneously identified ACE items with direct associations with providing regular care; for example, while living with anyone who was a problem drinker or alcoholic, or living with anyone who served time or was sentenced to serve time in prison, jail, or any other correctional facility remained statistically significant, living with anyone who used illegal drugs or abused prescription medication became insignificant, likely because this variable was mediated by the other two variables. Rerunning the model without these two ACE items resulted in the drug item being statistically significant (Odds Ratio = 1.18; 95% CI 1.06–1.31).

The role reversal in which a child assumes developmentally inappropriate adult roles and responsibilities for their age [1,2,3,4,5], resulting in caregiving behaviors that extend into adulthood [10], generally occurred for the different types of ACEs. With more ACEs came greater odds of providing regular care as adults. Direct and indirect ACE effects on providing regular care were identified. The largest association between the ACE items and providing regular care as an adult involved living with anyone who was mentally ill, a problem drinker, abused drugs, or was observed physically hurting their partner. Although the results found a comparatively small effect of parental marital status on providing regular care, which became insignificant after adjusting for the other ACE items, several studies have found that parentification is associated with divorce as the child takes on the role of caretaker for their parents or siblings as they face emotional stress and changes in family dynamics [1,19,31].

Duration of the ACE may influence the level of parentification, and parent divorce and separation could occur later in the child’s life, on average, than other ACE events. Unfortunately, our data does not contain the timing of the ACE. Further research may explore the long-term caregiving effect of parentification related to the timing of the ACE.

Third, we hypothesized that among those who provided regular care, it would be similar for each group versus parents as the number of types of ACEs increases. Contrary to the hypothesis, providing regular care for a spouse, child, sibling, or friend or non-relative compared with a parent was more common as the number of types of ACEs increased. In addition, as the number of types of ACEs increased, the lower odds of providing regular care for a parent versus one of these other groups increased. Parentification may result in anger and resentment towards parents [1,31], potentially explaining the inverse dose–response relationship between the number of types of ACEs and providing regular care for the parent group. Other possible mechanisms may include estrangement, geographic distance, parental mortality/illness trajectories, partner selection, “chosen family,” social network substitution, and socioeconomic constraints, as well as acknowledgement that differential opportunity to provide care may drive this pattern. Exploring these possible explanations deserves further study.

The greater odds of providing regular care for a friend or non-relative versus a parent as the number of types of ACEs increased was more pronounced than providing regular care for a spouse, child, or sibling versus a parent as the number of types of ACEs increased. While parentification causes the individual to better sense the emotional and practical needs of others [1,6], greater opportunity to care for friends or non-relatives may be a possible explanation for this result. Further research may address this observation.

### Limitations

The principal limitation of this study is that the design is cross-sectional with self-reported responses, retrospectively recalled ACEs, and caregiving questions anchored to the past 30 days, without intensity, duration, relationship quality, reciprocity, co-residence, or caregiver burden. This makes temporal ordering and causal interpretation particularly delicate; for example, current caregiving may influence reporting of distress, smoking or drinking, and may plausibly affect recall or willingness to endorse adversity items, while health status may confound both ACE reporting and caregiving propensity. Furthermore, “parentification” is not measured in these data but functions as an explanatory narrative rather than an empirically tested mediator. BRFSS does not include parentification measures and reference to parentification. Parentification is included in this paper for theoretical purposes and to offer alternative explanations (e.g., attachment processes, social support trajectories, socioeconomic continuity, adult relationship formation, or differential estrangement from parents following adversity). Finally, the study did not reflect data from the entire U.S. because only 13 areas asked questions about both ACEs and provision of regular care; yet, external validity is good to the extent that the decision to ask these questions in these areas was not influenced by higher levels of ACEs and providing regular care in these areas.

## 5. Conclusions

The study confirms a positive association between the number of ACEs and caregiving as adults. All the ACEs significantly positively influence providing regular care, with living with anyone who is depressed, mentally ill, or suicidal having the largest effect and having parents who are divorced having the smallest effect. However, in a model that simultaneously estimates all the types of ACEs, demographics, health and behavior variables, the only direct positive associations involve living with anyone who is depressed, mentally ill, or suicidal; living with anyone who is a problem drinker or alcoholic; living with anyone who served time or was sentenced to serve time in prison, jail, or any other correctional facility; having parents or adults in their home who physically hurt each other; and having anyone, at least 5 years older or an adult, ever touch you sexually or force you to have sex. Among those providing regular care, caring for a spouse, child, sibling, friend or non-relative is significantly greater than caring for a parent as the number of types of ACEs increases. This result is most pronounced for a friend or non-relative. There was no difference between providing regular care for a grandparent versus a parent as the number of types of ACEs increases. Why providing regular care as an adult is more likely for a spouse, child, sibling, or friend or non-relative versus a parent as the number of types of ACEs increase deserves further investigation. Several possible mechanisms for further study were mentioned.

While some caregiving behaviors are positive (empathy, responsibility, life skills, coping skills, giving, and autonomy), some consequences of parentification resulting in caregiving as adults may be negative (e.g., a tendency to prioritize others’ needs above one’s own, difficulty with saying “no” to requests, feeling responsible for others’ feelings, accepting poor treatment which may seem normal, difficulty delegating or trusting others, hypervigilance and emotional dysregulation). The negative consequences may also extend beyond the individual to family, friends, and future generations. Understanding that parentification helps explain the link between ACEs and providing regular caregiving as adults can help us identify the positive and negative aspects of ACE associated caregiving.

## Figures and Tables

**Table 1:** Number of participants and level of regular care or assistance by U.S. area and year.

		Complete Data		Regular Care or Assistance	
State	No.	No.	% *	% (SE) *	Year
Alabama	4007	3996	5.57	23.42 (0.89)	2021
Arkansas	4459	4438	3.06	25.30 (1.01)	2021
Florida	8616	8585	17.38	20.43 (1.06)	2020
Georgia	7106	7081	10.19	17.95 (0.71)	2020
Iowa	8081	8033	3.35	19.94 (0.57)	2021
Mississippi	4044	4032	3.40	25.53 (0.88)	2021
Nevada	4090	4076	6.22	20.06 (0.95)	2021, 2024
North Dakota	5350	5333	0.88	16.10 (0.65)	2021
Oregon	14,783	14,556	14.45	20.26 (0.43)	2021, 2022, 2023
South Carolina	7335	7297	4.79	24.56 (0.74)	2021
Tennessee	4642	4623	7.43	26.45 (0.93)	2023
Viginia	13,515	13,456	16.97	21.25 (0.53)	2022, 2024
Wisconsin	5165	5160	6.30	18.55 (0.77)	2021
Total	91,193	90,666	100.0		

Data source: BRFSS. * Weighted estimates, based on the complex sampling design. Complete data refers to those who responded “Yes” or “No” to the regular care or assistance variable.

**Table 5:** Odds of providing regular care or assistance for group X versus a parent, stepparent, or parent-in-law by number of types of ACEs.

			Model 1	Model 2	Model 3	Model 4	Model 5	Model 6
Distinct ACEs Experienced			Grandparent, step grandparent or grandparent-in-law	Spouse or partner	Child or stepchild, grandchild or step grandchild	Sibling, stepsibling, sibling-in-law, or other relatives	Friend or non-relative	Unknown
	No.	% (SE) *	Odds Ratio	Odds Ratio	Odds Ratio	Odds Ratio	Odds Ratio	Odds Ratio
			(95% CI) *^†^	(95% CI) *^†^	(95% CI) *^†^	(95% CI) *^†^	(95% CI) *^†^	(95% CI) *^†^
0	6241	27.96	1.00	1.00	1.00	1.00	1.00	1.00
1–2	6481	32.16	1.32	1.09	1.23	1.05	1.22	0.63
			(0.92–1.90)	(0.89–1.32)	(0.99–1.52)	(0.82–1.36)	(0.99–1.51)	(0.39–1.01)
3–4	3301	17.8	1.08	1.09	1.21	1.25	1.45	1.36
			(0.61–1.94)	(0.86–1.38)	(0.94–1.55)	(0.96–1.64)	(1.10–1.91)	(0.71–2.59)
≥5	3456	22.03	1.35	1.42	1.42	1.44	1.82	1.02
			(0.88–2.08)	(1.07–1.88)	(1.10–1.83)	(1.06–1.96)	(1.42–2.33)	(0.55–1.92)
Type 3 Analysis of Effects Pr > F			0.3015	0.1203	0.0432	0.0480	<0.0001	0.0814

Data source: BRFSS, 2020–2024. SE: Standard Error; CI: Confidence Interval. * Weighted estimates, based on the complex sampling design. ^†^ Adjusted for age, sex, race/ethnicity, marital status, education, annual household income, year, poor mental health days, poor physical health days, difficulty making decisions, depression, obesity, smoking status, and heavy drinking status.

## Data Availability

Data used in this study is publicly available through the CDCs BRFSS program.

## Abbreviations

The following abbreviations occur in this manuscript:

ACE	Adverse Childhood Experiences
BRFSS	Behavior Risk Factor Surveillance System
CDC	Centers for Disease Control and Prevention
CI	Confidence Interval
NH	Non-Hispanic
SE	Standard Error
SAS	Statistical Analysis System

## References

[1] Dariotis J.K., Chen F.R., Park Y.R., Nowak M.K., French K.M., Codamon A.M. (2023). Parentification vulnerability, reactivity, resilience, and thriving: A mixed methods systematic literature review. Int. J. Environ. Res. Public Health.

[2] Połomski P., Peplińska A., Lewandowska-Walter A., Borchet J. (2021). Exploring resiliency and parentification in Polish adolescents. Int. J. Environ. Res. Public Health.

[3] Burton L. (2007). Childhood adultification in economically disadvantaged families: A conceptual model. Fam. Relat..

[4] Hooper L.M., Levesque R.J.R. (2011). Parentificatio. Encyclopedia of Adolescence.

[5] Hooper L.M., Wallace S.A., Doehler K., Dantzler J. (2012). Parentification, ethnic identity, and psychological health in lack and White American college students: Implications of family-of-origin and cultural factors. J. Comp. Fam. Stud..

[6] Hendricks B.A., Vo J.B., Dionne-Odom J.N., Bakitas M.A. (2021). Parentification among young carers: A concept analysis. Child Adolesc. Soc. Work. J..

[7] Greenberg R., Jurkovic G.J. (1997). Lost Childhoods: The Plight of the Parentified Child.

[8] Jones R.A., Wells M. (1996). An empirical study of parentification and personality. Am. J. Fam. Ther..

[9] McMahon T.J., Luthar S.S. (2007). Defining characteristics and potential consequences of caretaking burden among children living in urban poverty. Am. J. Orthopsychiatry.

[10] Nuttall A.K., Coberly B., Diesel S.J. (2018). Childhood caregiving roles, perceptions of benefits, and future caregiving intentions among typically developing adult siblings of individuals with autism spectrum disorder. J. Autism Dev. Disord..

[11] Thompson M.J., Platts C.R., Davies P.T. (2024). Parent–child boundary dissolution and children’s psychological difficulties: A meta-analytic review. Psychol. Bull..

[12] Chen C.Y., Panebianco A. (2020). Physical and psychological conditions of parental chronic illness, parentification and adolescent psychological adjustment. Psychol. Health.

[13] Van Loon L.M., Van de Ven M.O., Van Doesum K.T., Hosman C.M., Witteman C.L. (2017). Parentification, stress, and problem behavior of adolescents who have a parent with mental health problems. Fam. Process.

[14] Burnett G., Jones R.A., Bliwise N.G., Ross L.T. (2006). Family unpredictability, parental alcoholism, and the development of parentification. Am. J. Fam. Ther..

[15] Hicks H.R. (2023). Experiences & long-term implications of childhood parentification & parental substance misuse: A scoping review. McNair Sch. Res. J..

[16] Fitzgerald M.M., Schneider R.A., Salstrom S., Zinzow H.M., Jackson J., Fossel R.V. (2008). Child sexual abuse, early family risk, and childhood parentification: Pathways to current psychosocial adjustment. J. Fam. Psychol..

[17] Pedersen S., Revenson T.A. (2005). Parental illness, family functioning, and adolescent well-being: A family ecology framework to guide research. J. Fam. Psychol..

[18] Peris T.S., Goeke-Morey M.C., Cummings E.M., Emery R.E. (2008). Marital conflict and support seeking by parents in adolescence: Empirical support for the parentification construct. J. Fam. Psychol..

[19] Jurkovic G.J., Thirkield A., Morrell R. (2001). Parentification of adult children of divorce: A multidimensional analysis. J. Youth Adolesc..

[20] Masiran R., Ibrahim N., Awang H., Lim P.Y. (2023). The positive and negative aspects of parentification: An integrated review. Child. Youth Serv. Rev..

[21] Blake-Holmes K., Maynard E., Brandon M. (2023). The impact of acquiescence: A model of coping developed from children of parents with mental illness. Adv. Ment. Health.

[22] Centers for Disease Control and Prevention (2013). The BRFSS User Guide. https://www.cdc.gov/brfss/data_documentation/pdf/UserguideJune2013.pdf.

[23] Centers for Disease Control and Prevention (2021). The Behavioral Risk Factor Surveillance System’s 2020 Summary Data Quality Report. https://www.cdc.gov/brfss/annual_data/2020/pdf/2020-sdqr-508.pdf.

[24] Centers for Disease Control and Prevention (2022). The Behavioral Risk Factor Surveillance System’s 2021 Summary Data Quality Report. https://www.cdc.gov/brfss/annual_data/2021/pdf/2021-DQR-508.pdf.

[25] Centers for Disease Control and Prevention (2023). The Behavioral Risk Factor Surveillance System’s 2022 Summary Data Quality Report. https://www.cdc.gov/brfss/annual_data/2022/pdf/2022-DQR-508.pdf.

[26] Centers for Disease Control and Prevention (2024). The Behavioral Risk Factor Surveillance System’s 2023 Summary Data Quality Report. https://www.cdc.gov/brfss/annual_data/2023/pdf/2023-DQR-508.pdf.

[27] Centers for Disease Control and Prevention (2025). The Behavioral Risk Factor Surveillance System’s 2024 Summary Data Quality Report. https://www.cdc.gov/brfss/annual_data/2024/pdf/2024-DQR-508.pdf.

[28] Centers for Disease Control and Prevention (2025). Behavioral Risk Factor Surveillance System Survey Data. https://www.cdc.gov/brfss/index.html.

[29] Castro D.M., Jones R.A., Mirsalimi H. (2004). Parentification and the impostor phenomenon: An empirical investigation. J. Fam. Ther..

[30] Gilford T.T. (2013). An Exploration of the Role of Perceived Social Support, Coping, and Resilience in the Academic Motivation of Parentified Black College Students. Doctoral Dissertation.

[31] Dragan M., Hardt J. (2016). Childhood adversities and risk for problematic alcohol use. Addict. Behav..

